# Supplementary material for: Clustering predicted structures at the scale of the known protein universe
Source: Nature. 2023 Sep 13;622(7983):637–45. doi: 10.1038/s41586-023-06510-w (PMC10584675; doi:10.1038/s41586-023-06510-w)
Supplement: Supplementary file 1 — Reporting Summary [file 41586_2023_6510_MOESM1_ESM.pdf]

nature portfolio

Corresponding author(s): Pedro Beltrao, Martin Steinegger

Last updated by author(s): Jul 13, 2023

Reporting Summary

Nature Portfolio wishes to improve the reproducibility of the work that we publish. This form provides structure for consistency and transparency in reporting. For further information on Nature Portfolio policies, see our Editorial Policies and the Editorial Policy Checklist.

Statistics

For all statistical analyses, confirm that the following items are present in the figure legend, table legend, main text, or Methods section.

n/a	Confirmed	
☐	☒	The exact sample size (*n*) for each experimental group/condition, given as a discrete number and unit of measurement
☒	☐	A statement on whether measurements were taken from distinct samples or whether the same sample was measured repeatedly
☐	☒	The statistical test(s) used AND whether they are one- or two-sided<br>*Only common tests should be described solely by name; describe more complex techniques in the Methods section.*
☒	☐	A description of all covariates tested
☐	☒	A description of any assumptions or corrections, such as tests of normality and adjustment for multiple comparisons
☐	☒	A full description of the statistical parameters including central tendency (e.g. means) or other basic estimates (e.g. regression coefficient) AND variation (e.g. standard deviation) or associated estimates of uncertainty (e.g. confidence intervals)
☐	☒	For null hypothesis testing, the test statistic (e.g. *F*, *t*, *r*) with confidence intervals, effect sizes, degrees of freedom and *P* value noted<br>*Give P values as exact values whenever suitable.*
☒	☐	For Bayesian analysis, information on the choice of priors and Markov chain Monte Carlo settings
☒	☐	For hierarchical and complex designs, identification of the appropriate level for tests and full reporting of outcomes
☒	☐	Estimates of effect sizes (e.g. Cohen's *d*, Pearson's *r*), indicating how they were calculated

Our web collection on statistics for biologists contains articles on many of the points above.

Software and code

Policy information about availability of computer code

Data collection	The structural clustering method is available at foldseek.com, is is implemented in Foldseek v4.645b789 and available as free and open-source software (GPLv3). MMseqs2/Linclust v14.7e284 is available at mmseqs.com.
Data analysis	The cluster analysis was performed using goatools v1.2.4 (https://github.com/tanghaibao/goatools), DeepFRI v0.0.1 for GO predictions (https://github.com/flatironinstitute/DeepFRI) ColabFold v1.5.2 for structure prediction (https://colabfold.com). For plotting Python v3.10.6 (https://www.python.org/), Matplotlib v3.6.2 (https://matplotlib.org/), seaborn v0.12.2 (https://github.com/mwaskom/seaborn), ChimeraX v1.5 (https://www.cgl.ucsf.edu/chimerax/), Pavian commit: cd2f21 (https://fbreitwieser.shinyapps.io/pavian/), pandas v1.5.2 (https:// github.com/pandas-dev/pandas) was used.

For manuscripts utilizing custom algorithms or software that are central to the research but not yet described in published literature, software must be made available to editors and reviewers. We strongly encourage code deposition in a community repository (e.g. GitHub). See the Nature Portfolio guidelines for submitting code & software for further information.

Data

Policy information about availability of data

All manuscripts must include a data availability statement. This statement should provide the following information, where applicable:

- Accession codes, unique identifiers, or web links for publicly available datasets
- A description of any restrictions on data availability
- For clinical datasets or third party data, please ensure that the statement adheres to our policy

Clustering data is freely and publicly available (CC-BY) at cluster.foldseek.com
All data generated and used for the analyses can be downloaded: https://afdb-cluster.steineggerlab.workers.dev

AlphaFold database v3 (https://alphafold.ebi.ac.uk/) was used for the analysis and is currently available at gs://public-datasets-deepmind-alphafold. For the analysis we used Pfam 34.0 (https://ftp.ebi.ac.uk/pub/databases/Pfam/releases/Pfam34.0), PDB (Oct 14th, 2022. https://www.rcsb.org), UniProt TrEMBL 2022_03 (https://ftp.ebi.ac.uk/pub/databases/uniprot/), SwissProt 2022_03 (https://ftp.ebi.ac.uk/pub/databases/uniprot/), ECOD 20230309 (http://prodata.swmed.edu/ecod/) and the MALISAM (http://prodata.swmed.edu/malisam/) database.

Research involving human participants, their data, or biological material

Policy information about studies with human participants or human data. See also policy information about sex, gender (identity/presentation), and sexual orientation and race, ethnicity and racism.

Reporting on sex and gender	Not applicable. The terms - sex and gender - were not used in the paper.
Reporting on race, ethnicity, or other socially relevant groupings	Not applicable. No social analysis is done in the paper.
Population characteristics	Not applicable. No human participant is included in the analysis
Recruitment	Not applicable. No participant is included in the analysis
Ethics oversight	Not applicable.

Note that full information on the approval of the study protocol must also be provided in the manuscript.

Field-specific reporting

Please select the one below that is the best fit for your research. If you are not sure, read the appropriate sections before making your selection.

☒ Life sciences ☐ Behavioural & social sciences ☐ Ecological, evolutionary & environmental sciences

For a reference copy of the document with all sections, see nature.com/documents/nr-reporting-summary-flat.pdf

Life sciences study design

All studies must disclose on these points even when the disclosure is negative.

Sample size	The entire size of AlphaFold2 Database v3 -214,684,311 protein structures - were used.
Data exclusions	We excluded fragmented proteins, singleton clusters and redundant protein through MMseqs2 clustering. Mainly, the analyses in the paper were done with the output of the process aforementioned - 2,302,908 clusters and 30,045,247 protein entries. But those which are excluded are depicted in the paper and provided in our data website (cluster.foldseek.com)
Replication	Not applicable. The outputs in the paper are done by computational methods. The computational method is deterministic so the result is identical for each replicate.
Randomization	Not applicable. We are not comparing across groups.
Blinding	Not applicable. We are not comparing across groups.

Reporting for specific materials, systems and methods

We require information from authors about some types of materials, experimental systems and methods used in many studies. Here, indicate whether each material, system or method listed is relevant to your study. If you are not sure if a list item applies to your research, read the appropriate section before selecting a response.

Materials & experimental systems

n/a	Involved in the study
☒	☐ Antibodies
☒	☐ Eukaryotic cell lines
☒	☐ Palaeontology and archaeology
☒	☐ Animals and other organisms
☒	☐ Clinical data
☒	☐ Dual use research of concern
☒	☐ Plants

Methods

n/a	Involved in the study
☒	☐ ChIP-seq
☒	☐ Flow cytometry
☒	☐ MRI-based neuroimaging
